# Sharing Big Data

**DOI:** 10.1107/S2052252516020364

**Published:** 2017-01-01

**Authors:** Marek Grabowski, Wladek Minor

**Affiliations:** a Department of Molecular Physiology and Biological Physics, University of Virginia, Charlottesville, VA 22903, USA

**Keywords:** Big Data, reproducibility, data sharing, metadata, open science

## Abstract

Macromolecular Big Data provide numerous challenges and a number of initiatives that are starting to overcome these issues are discussed.

Experimental reproducibility is the cornerstone of scientific research, upon which all progress rests. However, recent systematic surveys have revealed that a large fraction of representative sets of studies published in biomedical journals cannot be reproduced in another laboratory. This increased focus on reproducibility has likely contributed to the growing rate of retractions among scientific publications (Cokol *et al.*, 2008 ▸).

In contrast to many other areas of biomedical research, macromolecular crystallography has always been at the forefront of ‘reproducible research’ and ‘open science’, long before these approaches became widely appreciated and practiced. From the outset, crystallographers have embraced two fundamental tenets regarding crystallographic data: the preservation of relevant ‘data’ and making the data publicly available. Initially, the relevant data – the ‘D’ in the PDB (Protein Data Bank) – was limited to atomic coordinates, and was later supplemented by the ‘header’ containing the metadata describing the parameters of data collection (Berman *et al.*, 2000 ▸). Since the 1980s, deposition of structure coordinates into the PDB has been a requirement for the publication of a structure in scientific journals. As of 2006, structural deposits include structure factors, which permit recalculation of electron density maps. Yet the primary, ‘raw’ data of macromolecular crystallography, the sets of X-ray diffraction images used to derive the structure-factor files and atomic coordinates, typically have not been preserved, or if they have been preserved, have not been publicly available. In some cases, these data have been retained in ‘data silos’ reportedly kept by synchrotron facilities, individual crystallographers, or pharma companies. It is the experience of the authors of this commentary that only a very small fraction of requests for original diffraction images sent directly to authors of structures resulted in access to the data.

Traditionally, several factors have been regarded as very difficult challenges for creation of public repositories of diffraction data: (1) the sheer size of the data, which is 2–3 orders of magnitude greater than structure factors, (2) difficulties in organizing, acquiring, curating and managing the associated metadata, and (3) the deep-rooted tendency of researchers to keep their data private. Progress in storage technologies has almost eliminated the first challenge – the cost of hardware required to accommodate diffraction data has dropped significantly. The other two challenges still remain formidable, as discussed in the paper by Kroon-Batenburg, Helliwell, McMahon and Terwilliger in this issue of **IUCrJ** (Kroon-Batenburg *et al.*, 2017 ▸). The authors are long-term advocates of raw diffraction data preservation and are founding members of the Diffraction Data Working Group (DDWG). The paper presents an overview of several initiatives that have emerged in recent years to create public repositories of diffraction data and the diverse ways in which they approach these challenges.

The initiatives surveyed in the paper include dedicated diffraction data repositories such as the SBGrid (Meyer *et al.*, 2016 ▸) and the IRRMC (Grabowski *et al.*, 2016 ▸); institutional repositories such as the one run by the University of Manchester (Tanley, Schreurs, Kroon-Batenburg & Helliwell, 2012 ▸); general-purpose repositories for scientific data such as Zenodo and Research Gate; and synchrotron repositories such as the Synchrotron.Store (Meyer *et al.*, 2014 ▸). Between themselves, these resources now contain about 6600 publicly available diffraction datasets, corresponding to nearly 3600 diffraction experiments which have resulted in a PDB deposition. In addition, there are likely thousands more datasets sequestered in ‘dark data’, non-public data silos at synchrotron facilities or big pharma. Altering the traditional reluctance of researchers to share their data may best be addressed by funding-agency mandates stipulating that all data supporting publicly funded publications should be made publicly available. The two largest public repositories (Grabowski *et al.*, 2016 ▸, Meyer *et al.*, 2016 ▸) have used a variety of incentives to attract submissions from the community, amassing significant numbers of diffraction experiments (3118 and 253, respectively).

All this data would be, however, largely unusable without essential metadata, such as the identity of the sample and data collection parameters, which are necessary for optimal data reduction and structure determination. The latter is usually recorded in the headers of diffraction images – in one of the more than 200 formats defined by manufacturers of detectors and some synchrotron beamlines. The problem is that the metadata in the headers are sometimes missing, inconsistent, or just plain wrong. Herein lies the ‘metadata challenge’, common to many branches of science. A number of ongoing efforts are directed toward creating scientific ontologies and standardizing metadata (Ashburner *et al.*, 2000 ▸; Musen *et al.*, 2015 ▸). As the paper explains, structural biology has been in the vanguard of such efforts. The Crystallographic Information Framework (CIF) defined in the 1990s has created an ontology for structural biology (including the imgCIF dictionary for data collection) and defined a ‘holistic metadata framework for crystallography’. Unfortunately, as the authors note, perhaps with a bit of understatement, ‘not all real-world workflows use CIF as their actual mechanism for capturing data and metadata’.

The ultimate goal of an ideal repository is to provide a full description of diffraction experiment in the form that would allow others to easily perform data reduction and structure re-determination for every new deposit in the PDB. A re-examination of raw data and/or structure factors may bring a significant improvement in structure quality, as shown by the case of cisplatin-protein complexes discussed in the paper. In particular, a re-processing of the original diffraction data for cisplatin bound to hen egg lysozyme (Tanley, Schreurs, Kroon-Batenburg, Meredith *et al.*, 2012 ▸) accessible at the University of Manchester repository, and the subsequent re-refinement by a different group, improved the resolution of the crystal structure from 2.4 to 2.0 Å (Shabalin *et al.*, 2015 ▸) and a subsequent follow-up by the original group was able to improve resolution further to 1.7 Å (Tanley *et al.*, 2016 ▸). The ‘data debate’ which ensued in this case provides a nice illustration of the benefits of sharing data and trying different interpretations, even though, as the authors emphasize there is currently a lack of uniform community standards as to what is ‘the best’ interpretation. In this particular case, data sharing gave the same apparent improvement as could have been gained by performing new data collection on a hypothetical new powerful detector on a super-modern beamline and on a new generation synchrotron.

An often-overlooked Achilles heel of most biomedical databases and repositories is that negative results are often very well hidden or non-existent. The diffraction experiment that results in one protein–inhibitor complex is often the outcome of hundreds of diffraction data sets. The IRRMC has a mechanism that allows the deposition of data sets that did not bring expected results (for example an empty active site). For obvious reasons, such datasets not only have to be associated with a detailed description of the protein, but must also include which compounds were introduced and *via* which method. The negative information from screening results could identify ‘blind alleys’ and significantly speed-up drug discovery.

None of the current diffraction data repositories provides much, if any, information about the macromolecular sample. Ideally, one would like to have structural data integrated with data from expression, purification, and crystallization – as well as information about biomedical experiments. Creation of such integrated resources remains a dream that may be a major goal of BD2K type of program.
